# Application of target scanning combined with three-dimensional reconstruction in the diagnosis of early-stage lung adenocarcinoma

**DOI:** 10.7150/jca.92408

**Published:** 2024-09-23

**Authors:** Li Sun, Binbin Zhang, Pulin Li, Guanghe Fei, Ran Wang

**Affiliations:** Department of Respiratory and Critical Care Medicine, the First Affiliated Hospital of Anhui Medical University, Hefei 230022, China.

**Keywords:** early-stage lung adenocarcinoma, target scanning, three-dimensional reconstruction, diagnostic value, imaging techniques

## Abstract

**Objective:** This study aimed to assess the diagnostic value of target scanning combined with three-dimensional reconstruction in early-stage lung adenocarcinoma.

**Methods:** A retrospective analysis was conducted on 2017 patients with pathologically confirmed early-stage lung adenocarcinoma who underwent thoracoscopic lobectomy at the First Affiliated Hospital of Anhui Medical University from September 2018 to May 2023. These patients were initially diagnosed using conventional spiral CT scanning, and the study explored the application of target scanning combined with three-dimensional reconstruction in the diagnosis of early-stage lung adenocarcinoma.

**Results:** the pulmonary nodules were classified into three groups according to the pathological classification: Pre-Invasive lesion (PI), Microinvasive adenocarcinoma (MIA), and Invasive adenocarcinoma (IA), there were significant differences in the mean diameter of pulmonary nodules, the mean diameter of solid components, the proportion of solid components, pleural indentation, lobulation, spinous process, spiculation, and vascular convergence among the three groups. There were no significant differences between conventional spiral CT scanning and target scanning combined with three-dimensional reconstruction in terms of the number of cases with pure ground-glass nodules, mixed density nodules, pure solid nodules, the detection rate of vacuole signs, the CT value of the solid component and ground-glass component, and the maximum nodule diameter (P>0.05). However, target scanning combined with three-dimensional reconstruction detected a higher number of cases with lobulation signs, spinous process signs, pleural depression signs, burr signs, vessel convergence signs, and larger maximum diameters of the solid component compared to conventional spiral CT scanning (P<0.05).

**Conclusions:** Target scanning combined with three-dimensional reconstruction provides more reliable imaging evidence for the diagnosis of early-stage lung adenocarcinoma, particularly in identifying specific signs and characterizing solid components.

## Introduction

Lung cancer is a significant public health concern in China, with the highest incidence and mortality rates in 2022, as reported in the National Cancer Report^1^. Lung adenocarcinoma represents the most common pathological subtype of lung cancer^2^. The World Health Organization classifies lung adenocarcinoma into distinct stages, including atypical adenomatous hyperplasia (AAH), adenocarcinoma in situ (AIS), minimally invasive adenocarcinoma (MIA), and invasive adenocarcinoma (IAC)^3^. The 5-year survival rate after resection of AIS was 100%, and the 5-year survival rate of MIA was closed to 100%^4^, ^5^, early-stage lung cancer often lacks evident clinical symptoms or laboratory abnormalities, necessitating reliance on CT examinations for detection^6^. Low-dose spiral CT has been a common tool for early lung cancer screening, but it often results in a high false-positive rate^7^, ^8^. In contrast, the target scanning technique offers advantages such as thin slices, small pixel size, improved spatial resolution, and enhanced visualization of lesion characteristics and margins^9^. Moreover, it facilitates rapid image processing and three-dimensional reconstruction, enabling detailed anatomical examination and multi-angle observation of small lesions. This approach holds promise for improving early lung cancer diagnosis rates.

The aim of this study is to assess the diagnostic value of target scanning combined with three-dimensional reconstruction in early-stage lung adenocarcinoma.

## Materials and methods

A retrospective analysis was conducted on a cohort of 2017 patients diagnosed with early-stage lung adenocarcinoma who underwent thoracoscopic lobectomy from July 2018 to May 2023. All patients underwent both routine CT scanning and target scanning combined with 3D reconstruction before the operation. The inclusion criteria encompassed patients with a confirmed diagnosis of early lung adenocarcinoma by pathology, willingness to undergo surgery, availability of complete medical records, and clear CT imaging data. Exclusion criteria comprised patients with a survival prognosis of fewer than 3 months, individuals with mental disorders or cognitive impairment, those with concurrent pulmonary diseases (e.g., tuberculosis, pneumonia, bronchitis), patients with lung metastasis originating from other tumors, those with poor compliance or inadequate imaging data, as well as individuals suffering from severe heart or kidney diseases. Ethical approval for this study was obtained from the Medical Ethics Committee.

### Imaging protocol

All patients were subjected to imaging using a GE 128-slice spiral CT scanner. Initial chest scans were performed with a tube voltage of 120 kV, tube current ranging from 47 to 59 mAs, a slice thickness of 5 mm, collimation of 128 mm × 0.6 mm, and a slice distance of 1.2 mm. Subsequently, when pulmonary nodules were identified, thin-section high-resolution target scans with a narrow FOV (field of view) of 20 cm, and slice thicknesses of 1 mm and 0.8 mm, were conducted. The acquired images were then uploaded to a post-processing workstation for 3D reconstruction, including surface reconstruction, volume reconstruction, multi-plane reconstruction, and minimum density projection.

### Data analysis

CT images were retrospectively analyzed by a chief physician and a senior attending physician. The counted data encompassed the presence or absence of specific signs, including lobulated sign, spiculate sign, air bronchogram, vacuole sign, vessel convergence, and pleura traction. Measurement data consisted of CT values of the nodule, CT values of the solid component, CT values of the ground glass component, the CT value of ground-glass nodule was measured in Hounsfield Units (HU). The maximum nodule diameter, and maximum diameter of the solid component, the maximum diameter of ground-glass nodule was measured in centimetre(cm).

### Statistical analysis

Statistical analysis was performed to compare the characteristics of nodules obtained by routine scanning with those obtained by target scanning combined with 3D reconstruction. SPSS 22.0 was used as the statistical tool. Count data were expressed as (n, %), and the chi-squared (Χ²) test was employed for comparison. A p-value of < 0.05 was considered indicative of statistical significance.

## Results

### Patient demographics

**Figure 1:**
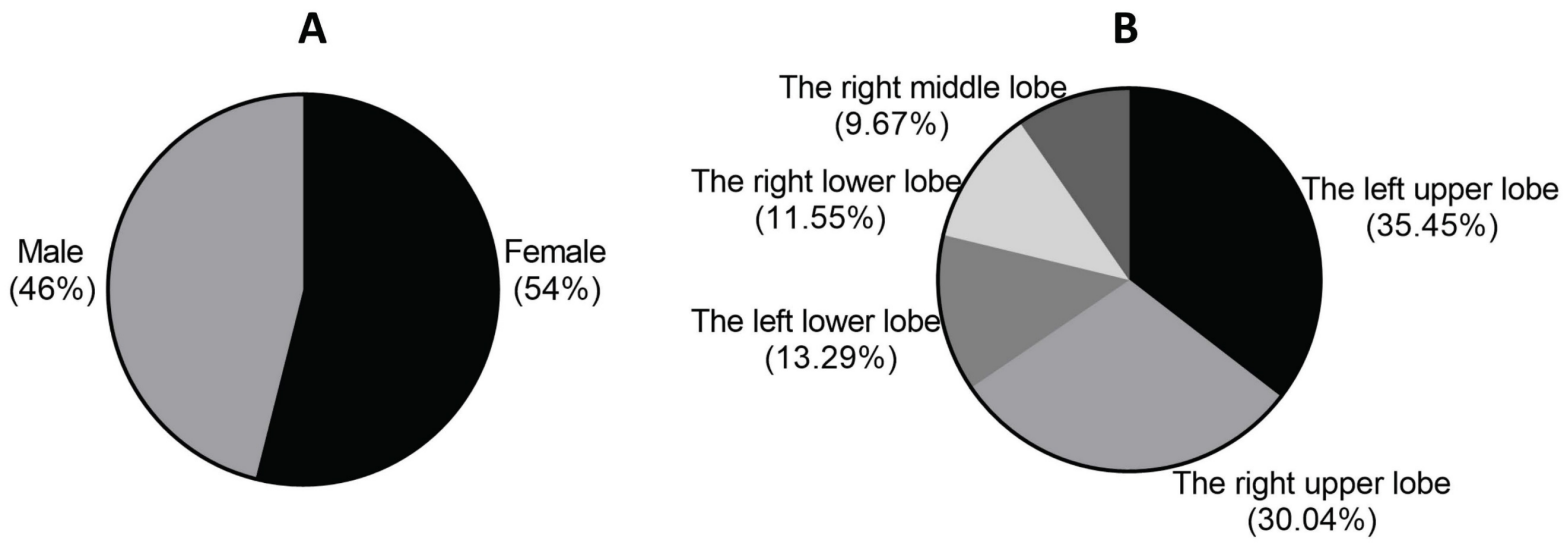
Gender distribution and lesion location in 2017 patients with early-stage lung adenocarcinoma.

Among the 2,017 patients, 1,088 were female and 929 were male, with only 206 having a history of smoking. The age distribution ranged from 27 to 83 years. In terms of lobes, there were 715 patients in the left upper lobe, 606 in the right upper lobe, 268 in the left lower lobe, 233 in the right lower lobe, and 195 cases in the right middle lobe. The maximum diameter of pulmonary nodules ranged from 0.5cm to 3cm (Figure 1).

### Imaging features and pathological staging

The pulmonary nodules were classified into three groups according to the pathological classification: Pre-Invasive lesion (PI), Microinvasive adenocarcinoma (MIA), and Invasive adenocarcinoma (IA). There were significant differences in the mean diameter of pulmonary nodules, the mean diameter of solid components, the proportion of solid components, pleural indentation, lobulation, spinous process, spiculation, and vascular convergence among the three groups (p < 0.05). However, there was no significant difference in vacuole proportions among the three groups (P > 0.05) (Table 1, Table 2).

### Imaging features between conventional spiral CT and target scan combined with 3D reconstruction

In conventional spiral CT, there were 373 cases (18.49%) of pure ground-glass nodules, 277 cases (13.73%) of pure solid nodules, and 1367 cases (67.77%) of partial solid nodules. In the target scan combined with 3D reconstruction, there were 203 cases (10.06%) of pure ground-glass nodules, 183 cases (9.07%) of pure solid nodules, and 1631 cases (80.86%) of mixed ground-glass nodules. The detection rate of different types of pulmonary nodules did not show a significant difference between conventional spiral CT and target scan combined with 3D reconstruction (Table 3).

### 3.4 Morphological signs between conventional spiral CT and target scan combined with 3D reconstruction

In conventional spiral CT, there were 368 cases of pleural indentation, 1261 cases of lobulation, 987 cases of spinous process, 1210 cases of spiculation, 356 cases of vacuole, and 845 cases of vascular convergence. Conversely, in the target scan combined with 3D reconstruction, there were 631 cases of pleural indentation, 1794 cases of lobulation, 1429 cases of spinous process, 1763 cases of spiculation, 428 cases of vacuole, and 1492 cases of vessel convergence. The detection rate of pleural indentation, lobulation, spinous process, spiculation, and vascular convergence was significantly higher in the target scan combined with 3D reconstruction compared to conventional spiral CT. However, there was no significant difference in the detection rate of the vacuole sign between the two groups (Table 4).

### 3.5 Comparison of the CT value, maximum diameter and solid components of ground-glass nodule of conventional spiral CT and target scan combined with 3D reconstruction

**Figure 2:**
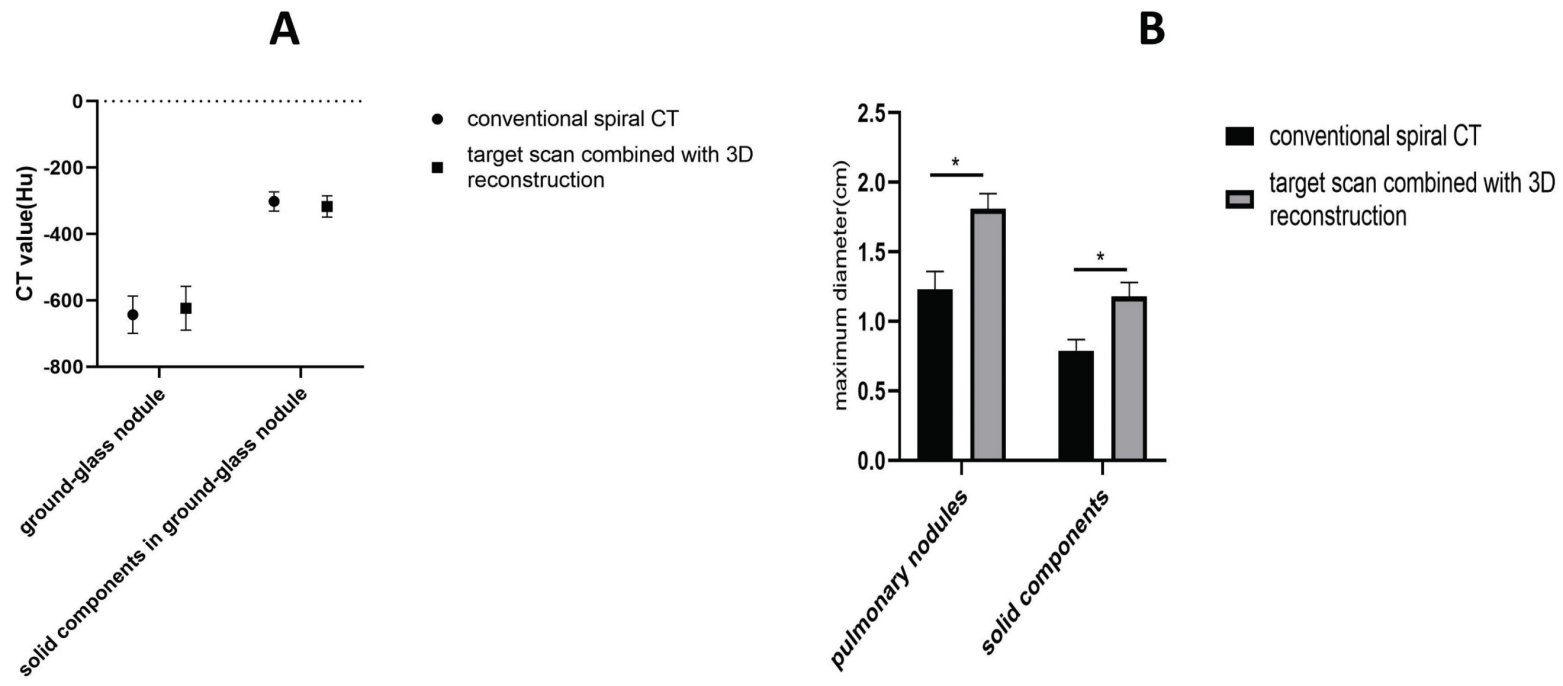
A. Comparison of the CT value (Hu) of ground-glass nodule and solid components of conventional spiral CT and target scan combined with 3D reconstruction in 2017 patients with early-stage lung adenocarcinoma. B. Comparison of the maximum diameter(cm) of pulmonary nodule and solid components of conventional spiral CT and target scan combined with 3D reconstruction in 2017 patients with early-stage lung adenocarcinoma.

There was no significant difference in the CT value of ground-glass nodules and the CT value of solid components within ground-glass nodules between conventional spiral scan and target scan combined with 3D reconstruction. However, the maximum diameter of pulmonary nodules and the maximum diameter and proportion of solid components, as measured by target scan combined with 3D reconstruction, were significantly higher than those measured by conventional spiral CT. These differences were found to be statistically significant (Figure 2).

## Discussion

Lung cancer is the leading cause of morbidity and mortality in China. Patients with lung cancer generally have a poor prognosis. The imaging findings of invasive pulmonary adenocarcinoma (IP) and minimally invasive adenocarcinoma (MIA) are similar, mostly presenting as pure ground-glass nodules with a slow growth cycle. The volume doubling time (VDT) often exceeds 800 days. The 5-year disease-free survival (DFS) for adenocarcinoma in situ (AIS) is 100%, while it is also 100% or nearly 100% for MIA. Diagnosing early-stage lung cancer is crucial as there are no typical clinical manifestations or abnormal laboratory indexes in the early stage^10^. Target scanning can effectively visualize small pulmonary lesions, improving the resolution and clarity of conventional spiral CT scans. Three-dimensional reconstruction has the advantage of displaying the shape, lobulation sign, spinous process sign, pleural depression sign, burr sign, and vessel convergence sign of lung nodules. It compensates for the low spatial resolution of CT tomographic images, thus excluding suspicious lesions^8^. The results of this study showed no significant difference in the detection rate of pure ground-glass nodules, mixed density nodules, pure solid nodules, the presence of vacuole sign, and the CT value of the solid and ground-glass components between conventional spiral CT scanning and target scanning combined with three-dimensional reconstruction. However, the detection rate of lobulated sign, spinous process sign, pleural indentation sign, spiculation sign, and vessel convergence sign was significantly higher with target scanning combined with three-dimensional reconstruction compared to conventional spiral CT scanning. Moreover, the CT reconstructed scan's contrast-to-noise ratio (CTR) was higher than that of conventional spiral CT scanning^11^, ^12^.

The lobulation sign is attributed to the varying growth speed and differentiation degree of the tumor as well as the constraints posed by surrounding structures^13^. When examined under a pathological microscope, lobulated notches reveal pulmonary stent structures such as vessels and hyperplastic fibrous tissue. Benign nodules typically have well-defined and smooth margins without noticeable lobulation, although some benign solitary pulmonary nodules may exhibit lobules, such as tuberculomas or chronic inflammatory nodules. Most of these lesions display superficial lobulation. Thus, the presence of deep lobulation and spiculation is of considerable importance in distinguishing lung cancer^14^. This study's findings demonstrate that target scanning combined with three-dimensional reconstruction exhibits a higher detection rate of the lobulation sign compared to conventional scanning.

The presence of the burr sign is caused by the extension and infiltration of the tumor into the adjacent lung tissue, reflecting direct tumor invasion^15^. Research studies have consistently demonstrated a higher occurrence of burrs, particularly dense short burrs, in malignant nodules compared to benign ones. Our findings also indicate a significantly higher detection rate of the burr sign in early-stage lung adenocarcinoma when using target scanning combined with three-dimensional reconstruction rather than conventional scans.

The pleural depression sign, characterized by localized pleural invagination resulting from pulmonary lesions, is caused by the contraction of fibrous scars in the nodule and traction of the adjacent visceral pleura. This creates a horn-shaped indentation, as evidenced by CT scans showing the tip pointing to the lesion and linear shadows connected to it. Research has established the pleural depression sign as a powerful indicator for differentiating between benign and malignant nodules. The incidence of pleural indentation in malignant nodules is approximately 58%^16^. In line with these findings, our results highlight the high detection rate of the pleural depression sign when using target scanning combined with three-dimensional reconstruction.

The vessel convergence sign refers to the increase, elongation, and aggregation of blood vessels around lung nodules. This occurs when malignant tumor tissue infiltrates the adjacent broncho-vascular bundles and interlobular septa or stimulates the proliferation of fibrous components, leading to the stretching of surrounding structures and causing the blood vessels in the vicinity to become twisted, rigid, or aggregated. As tumor infiltration and metabolic activity increase, the tumor's blood supply vessels thicken. In the early stages of lung cancer, the vessels around the tumor tend to grow toward the focal point or form new tumor vessels through budding. Abnormal changes in the perifocal vessels should raise suspicions of malignancy.

Target scanning offers several advantages including high contrast resolution and high structure resolution. Additionally, physiological ventilatory assistance positions the lesion at the highest point of the body's gravity line, facilitating greater gas entry into the lesion area. This causes overinflation of the lungs in the lesion area and ensures full extension of both the lesion and its background lung structure. These benefits are invaluable for studying biomechanics and anatomy, such as the lobular airway, blood vessels, lobules, septa, pulmonary interstitial, and pulmonary nodules 9. When evaluating the internal density of lung nodules, there is no significant difference in the recognition of lesion densities (pure ground-glass nodules, mixed density nodules, pure solid nodules) due to the use of three-dimensional reconstructions derived from TSCT scans that all present gray-scale contrast images. However, the introduction of target scans, improved scanning conditions, and reduced irradiation field have enhanced the density resolution of the images, allowing for clearer observation of the internal structure of the lesions. Notably, for semi-solid nodules with half ground-glass opacity, target scans demonstrate higher sensitivity than conventional spiral scans in detecting solid components within the nodules. Both lobulated sign and spiculate sign are well-recognized morphological changes indicating malignancy in lung cancer. However, these signs may be subtle during the early stages of malignant growth in lung adenocarcinoma, and the volume of the nodules may not be clearly evident. Three-dimensional reconstruction allows for the observation of lesion features from various directions and angles, revealing subtle changes that cannot be detected with a single visual angle. Changes in lung cancer affecting the small airways and bronchi can be manifested in four ways: bronchial truncation, invasion and narrowing of the bronchus, thickening of the bronchial wall, stenosis of the lumen, and stretching and dilation of the bronchus. It is challenging to observe bronchial changes within the focus due to cross sections being perpendicular or at certain angles to the shape of the bronchus. However, three-dimensional reconstruction provides a significant advantage in observing changes in the small airways within the focus.

Typical pleural indentation refers to a group of pleural indentation changes with distinct morphological alterations observed on spiral thin-layer scan. These changes typically entail a prominent horn shadow at the center of the indentation. By adjusting the angle for optimal observation, the pleural indentation sign can be effectively assessed, particularly in relation to pleural thickening and adhesion. When combined with three-dimensional reconstruction, target scanning significantly enhances the visualization of morphological characteristics of pulmonary nodules. It also allows for the evaluation of nodule edges and infiltration, exploration of internal nodule structures, assessment of blood vessel growth, and calculation of volume doubling time. Accurate diagnosis of pulmonary nodules through target scanning is valuable for the early detection of lung cancer. Micropapil-lary-predominant lung adenocarcinomas and solid infiltrating adenocarcinoma usually display as round solid nodules. Most of them are located in the upper lobes of both lungs, with clear boundary. The signs of lobulation, prick, vascular change and pleural depression are common, while the signs of air bronchus and vacuole are rare.

### Limitations

This study has a few local limitations. Firstly, CT target scans are more prone to artifacts and are susceptible to the effects of heartbeat and breathing movements. Consequently, small pulmonary nodules located near the chest membrane and heart may not be clearly visible on CT scan images. Secondly, CT target scanning necessitates two scans, resulting in increased burden on the patients. Thirdly, there may be variations in the evaluation of radiographs and pathological radiographs among radiologists and pathologists. Fourthly, the study did not stratify cases by relevant indicators such as sex and age. Finally, this study is based on single-center data, and the collection of multi-center data could be beneficial for further analysis.

## Figures and Tables

**Table 1:** Analysis results of imaging features and pathological staging in 2017 patients with early-stage lung adenocarcinoma.

		PI (n=431)	MIA (n=528)	IA (n=1058)
Pleural Indentation	Conventional spiral CT	153 (35.5%)	137 (25.95%)	78 (7.37%)
	Target scan combined with 3D reconstruction	287 (66.59%)	204 (38.64%)	140 (13.23%)
Lobulation	Conventional spiral CT	275 (63.81%)	266 (50.38%)	720 (68.05%)
	Target scan combined with 3D reconstruction	368 (85.38%)	471 (89.20%)	955 (90.26%)
Spinous Process	Conventional spiral CT	214 (49.65%)	457 (86.55%)	316 (29.87%)
	Target scan combined with 3D reconstruction	337 (78.19%)	503 (95.27%)	589 (55.67%)
Spiculation	Conventional spiral CT	239 (55.45%)	314 (59.47%)	657 (63.80%)
	Target scan combined with 3D reconstruction	413 (95.82%)	479 (90.72%)	871 (82.33%)
Vacuole	Conventional spiral CT	73 (16.94%)	104 (19.7%)	179 (16.92%)
	Target scan combined with 3D reconstruction	96 (22.27%)	147 (27.84%)	185 (17.49%)
Vascular Convergence	Conventional spiral CT	364 (84.45%)	273 (51.70%)	208 (19.66%)
	Target scan combined with 3D reconstruction	413 (95.82%)	385 (72.92%)	694 (65.60%)

**Table 2:** Analysis results of the mean diameter, the proportion of solid components and pathological staging in 2017 patients with early-stage lung adenocarcinoma.

		PI (n=431)	MIA (n=528)	IA (n=1058)
Mean diameter of pulmonary nodules (cm)	Conventional spiral CT	0.52±0.17	0.75±0.2	1.63±0.56
	Target scan combined with 3D reconstruction	0.67±0.18	0.94±0.42	2.41±0.89
Mean diameter of solid components(cm)	Conventional spiral CT	0.13±0.07	0.28±0.11	1.17±0.43
	Target scan combined with 3D reconstruction	0.22±0.12	0.37±0.14	1.52±0.60
Proportion of solid components (%)	Conventional spiral CT	25%	37.33%	71.78%
	Target scan combined with 3D reconstruction	32.84%	39.36%	63.07%

**Table 3:** Analysis results of the detection rate of different types of pulmonary nodules with conventional spiral CT and target scan combined with 3D reconstruction in 2017 patients with early-stage lung adenocarcinoma.

	Pure ground-glass nodules	Pure solid nodules	Partial solid nodules
Conventional spiral CT	373(18.49%)	277(13.73%)	1367(67.77%)
Target scan combined with 3D reconstruction	203(10.06%)	183(9.07%)	1631(80.86%)

**Table 4:** Analysis results of morphological signs with conventional spiral CT and target scan combined with 3D reconstruction in 2017 patients with early-stage lung adenocarcinoma.

	Pleural indentation	Lobulation	Spinous process	Spiculation	Vacuole	Vascular convergence
Conventional spiral CT	368 (18.24%)	1261 (62.52%)	987 (48.93%)	1210 (59.99%)	356 (17.65%)	845 (41.89%)
Target scan combined with 3D reconstruction	631 (31.28%)	1794 (88.94%)	1429 (70.85%)	1763 (87.41%)	428 (21.22%)	1492 (73.97%)
